# Supplementary material for: A yeast phenomic model for the influence of Warburg metabolism on genetic buffering of doxorubicin
Source: Cancer Metab. 2019 Oct 23;7:9. doi: 10.1186/s40170-019-0201-3 (PMC6806529; doi:10.1186/s40170-019-0201-3)
Supplement: Supplementary file 9 — Additional file 9. GO term-specific heatmaps for REMc/GTF-enriched clusters. GO term-specific heatmaps for significant GO process terms were generated as described in methods and Figs. 3 and 4. Any related child terms are presented in subsequent pages of the parent file name. GO terms with more than 100 children, with 2 or fewer genes annotated to the term, or a file size over 300KB are not shown. All heatmaps are generated with the same layout (see Figs. 3 and 4). [file 40170_2019_201_MOESM9_ESM.bz2 › Additional_File9_GOTermHeatmaps/Additional_File9_GOTermHeatmap/snoRNA_metabolic_process.pdf]

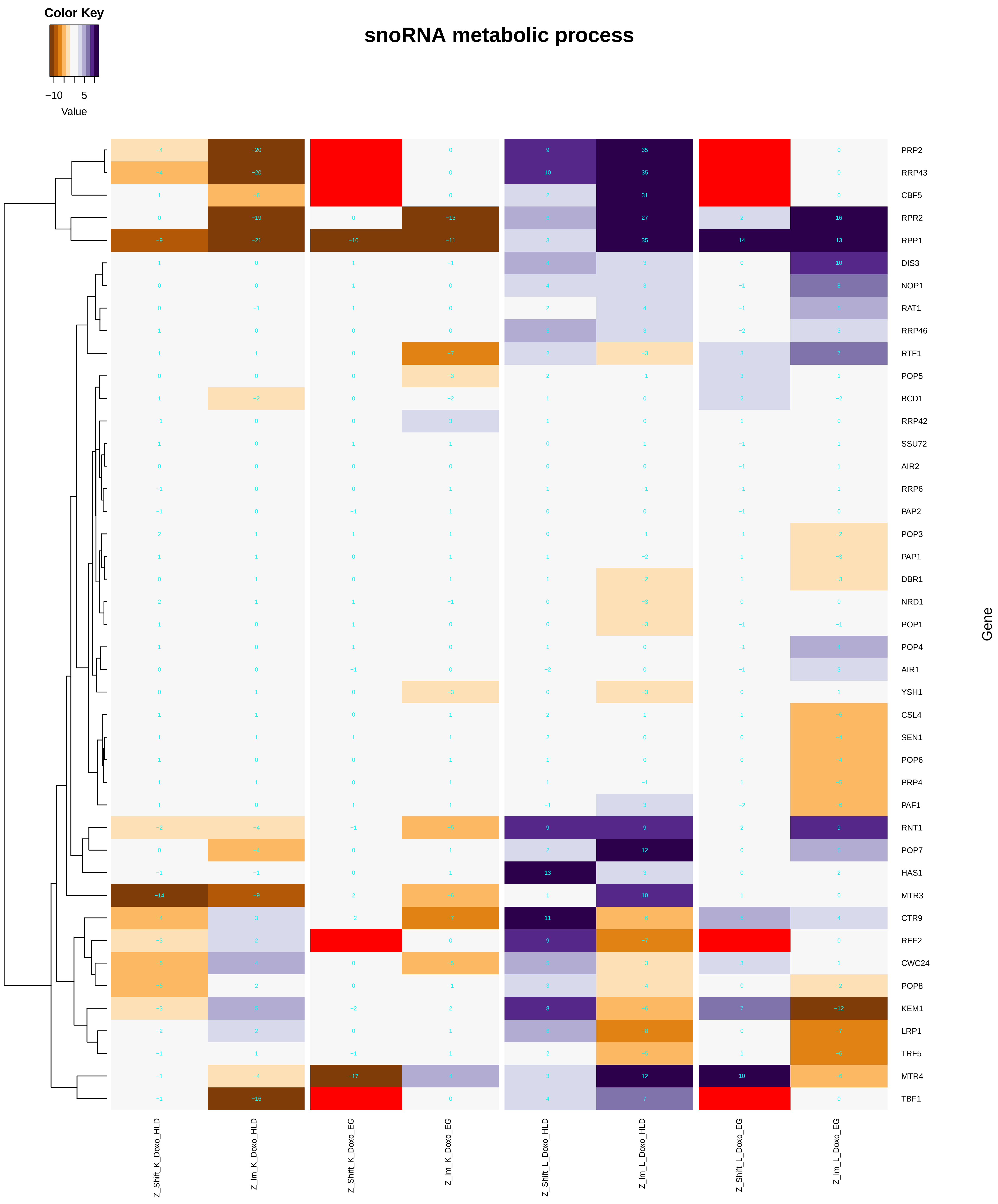
Color Key
−10 5
Value
snoRNA metabolic process
PRP2
RRP43
CBF5
RPR2
RPP1
DIS3
NOP1
RAT1
RRP46
RTF1
POP5
BCD1
RRP42
SSU72
AIR2
RRP6
PAP2
POP3
PAP1
DBR1
NRD1
POP1
POP4
AIR1
YSH1
CSL4
SEN1
POP6
PRP4
PAF1
RNT1
POP7
HAS1
MTR3
CTR9
REF2
CWC24
POP8
KEM1
LRP1
TRF5
MTR4
TBF1
Gene
Z_Shift_K_Doxo_HLD
Z_Im_K_Doxo_HLD
Z_Shift_K_Doxo_EG
Z_Im_K_Doxo_EG
Z_Shift_L_Doxo_HLD
Z_Im_L_Doxo_HLD
Z_Shift_L_Doxo_EG
Z_Im_L_Doxo_EG

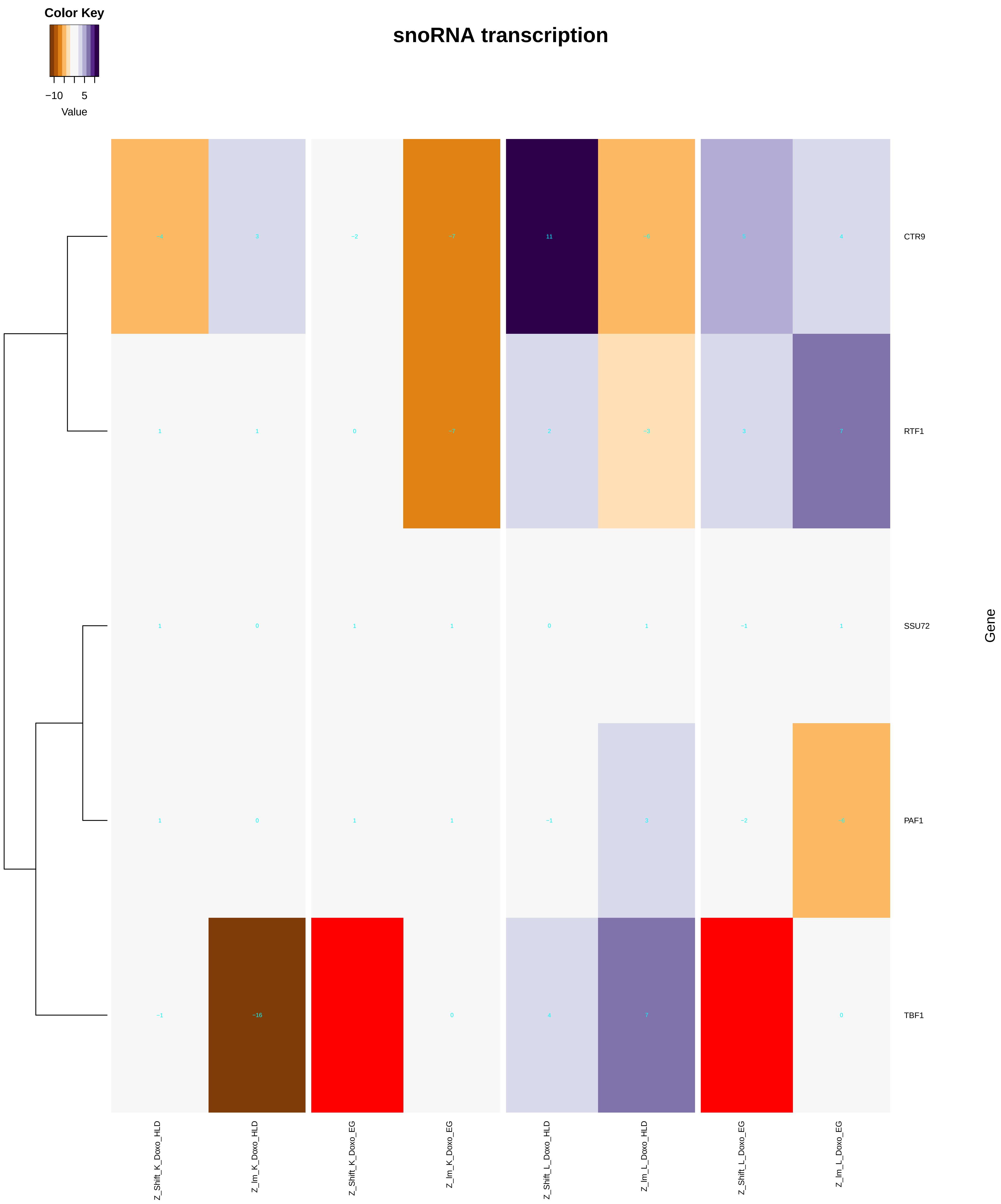

Color Key
−10
5
Value
snoRNA transcription
CTR9
RTF1
SSU72
PAF1
TBF1
Gene
Z_Shift_K_Doxo_HLD
Z_Im_K_Doxo_HLD
Z_Shift_K_Doxo_EG
Z_Im_K_Doxo_EG
Z_Shift_L_Doxo_HLD
Z_Im_L_Doxo_HLD
Z_Shift_L_Doxo_EG
Z_Im_L_Doxo_EG

snoRNA catabolic process

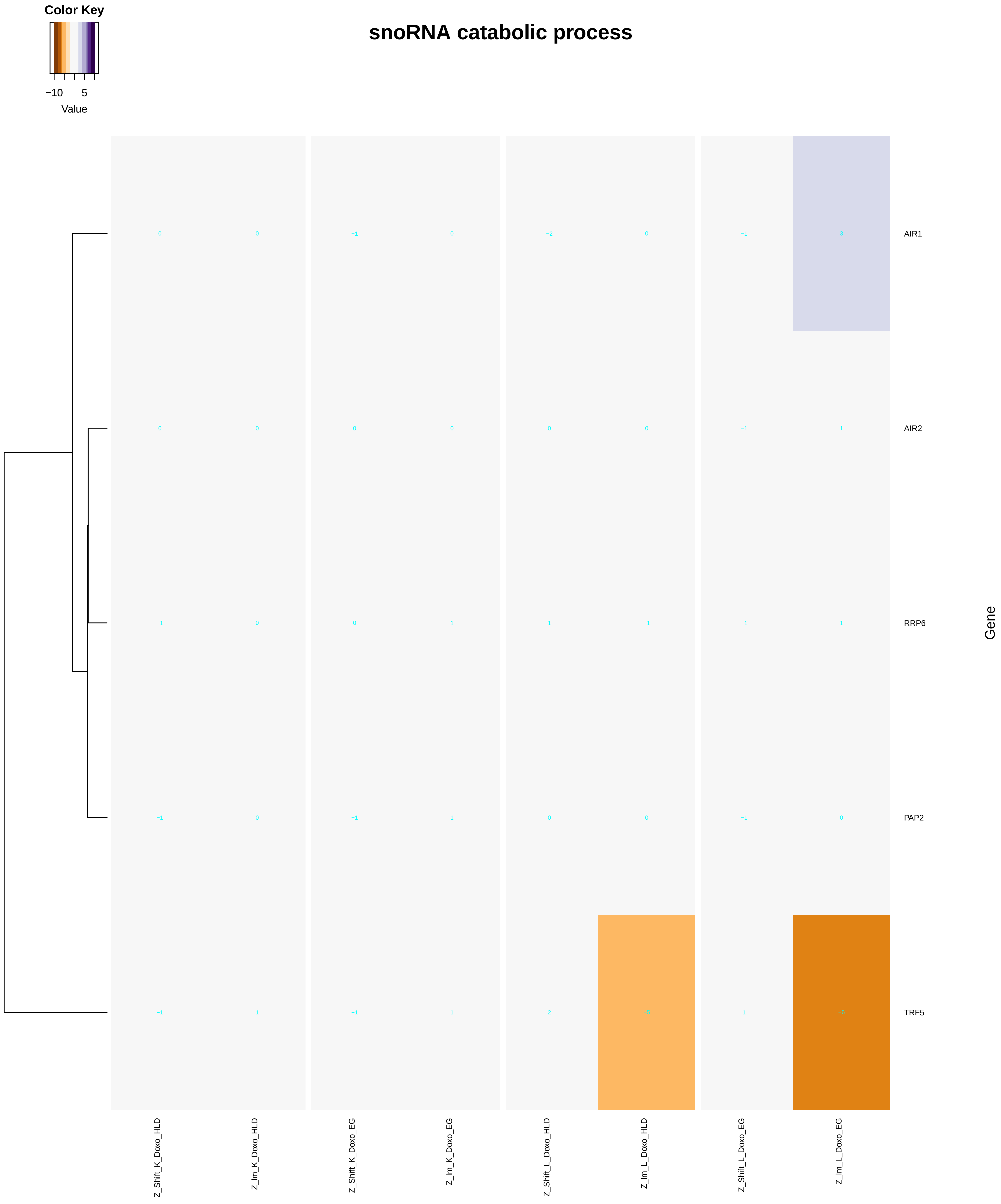

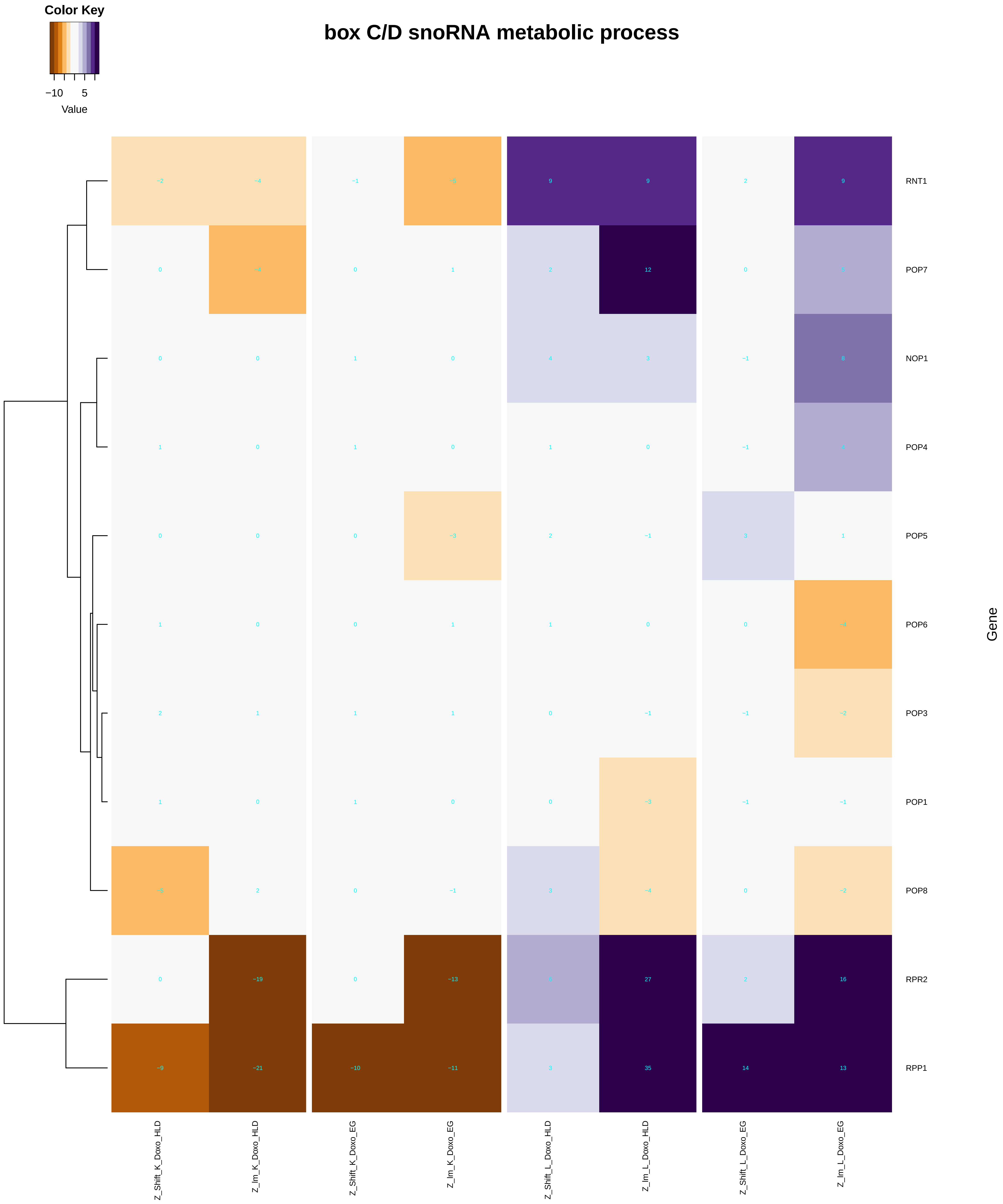
box C/D snoRNA metabolic process
Color Key
−10
5
Value
RNT1
POP7
NOP1
POP4
POP5
POP6
POP3
POP1
POP8
RPR2
RPP1
Gene
Z_Shift_K_Doxo_HLD
Z_lm_K_Doxo_HLD
Z_Shift_K_Doxo_EG
Z_lm_K_Doxo_EG
Z_Shift_L_Doxo_HLD
Z_lm_L_Doxo_HLD
Z_Shift_L_Doxo_EG
Z_lm_L_Doxo_EG

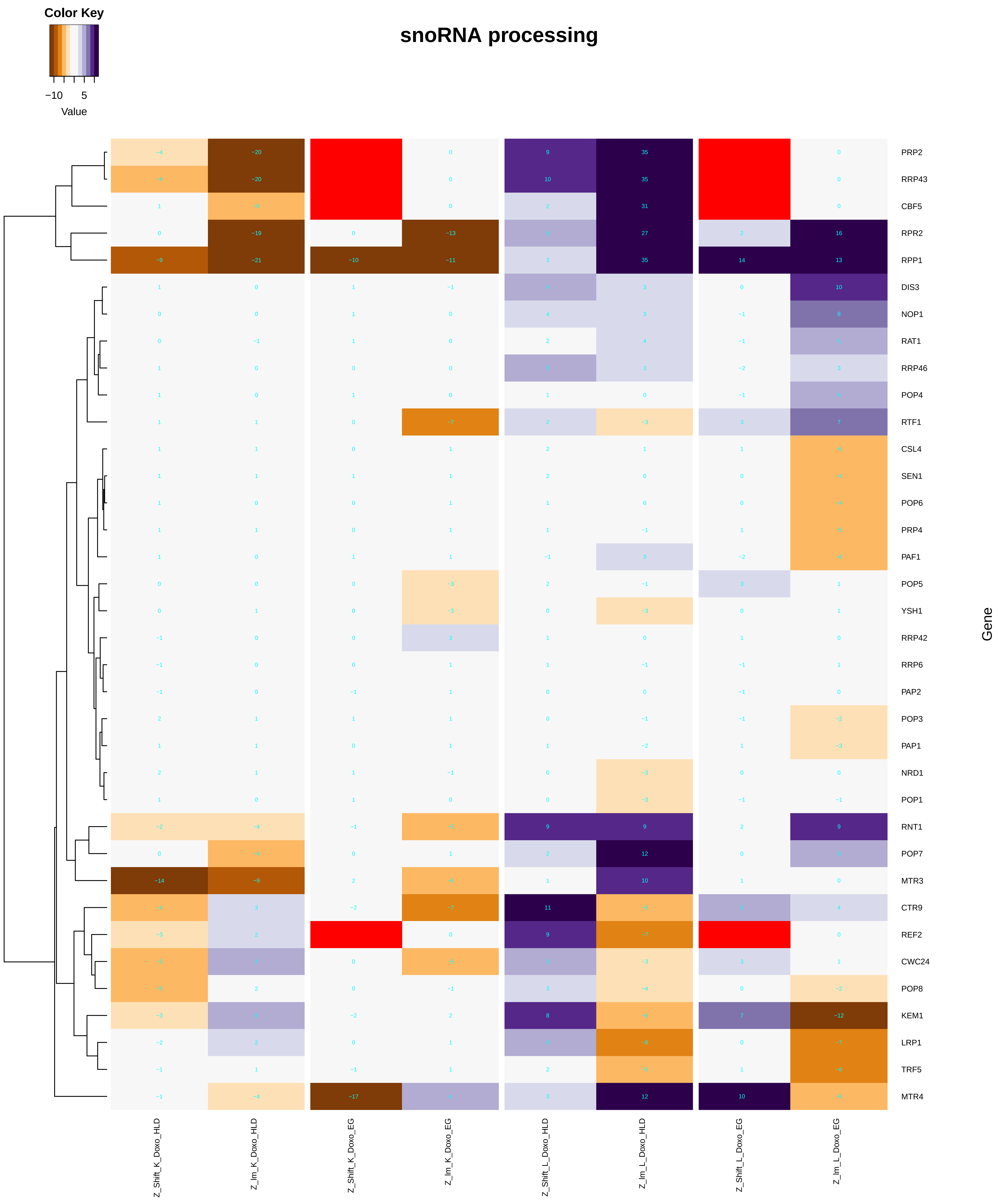
snoRNA processing
Color Key
−10
5
Value
PRP2
RRP43
CBF5
RPR2
RPP1
DIS3
NOP1
RAT1
RRP46
POP4
RTF1
CSL4
SEN1
POP6
PRP4
PAF1
POP5
YSH1
RRP42
RRP6
PAP2
POP3
PAP1
NRD1
POP1
RNT1
POP7
MTR3
CTR9
REF2
CWC24
POP8
KEM1
LRP1
TRF5
MTR4
Gene
Z_Shift_K_Doxo_HLD
Z_Im_K_Doxo_HLD
Z_Shift_K_Doxo_EG
Z_Im_K_Doxo_EG
Z_Shift_L_Doxo_HLD
Z_Im_L_Doxo_HLD
Z_Shift_L_Doxo_EG
Z_Im_L_Doxo_EG

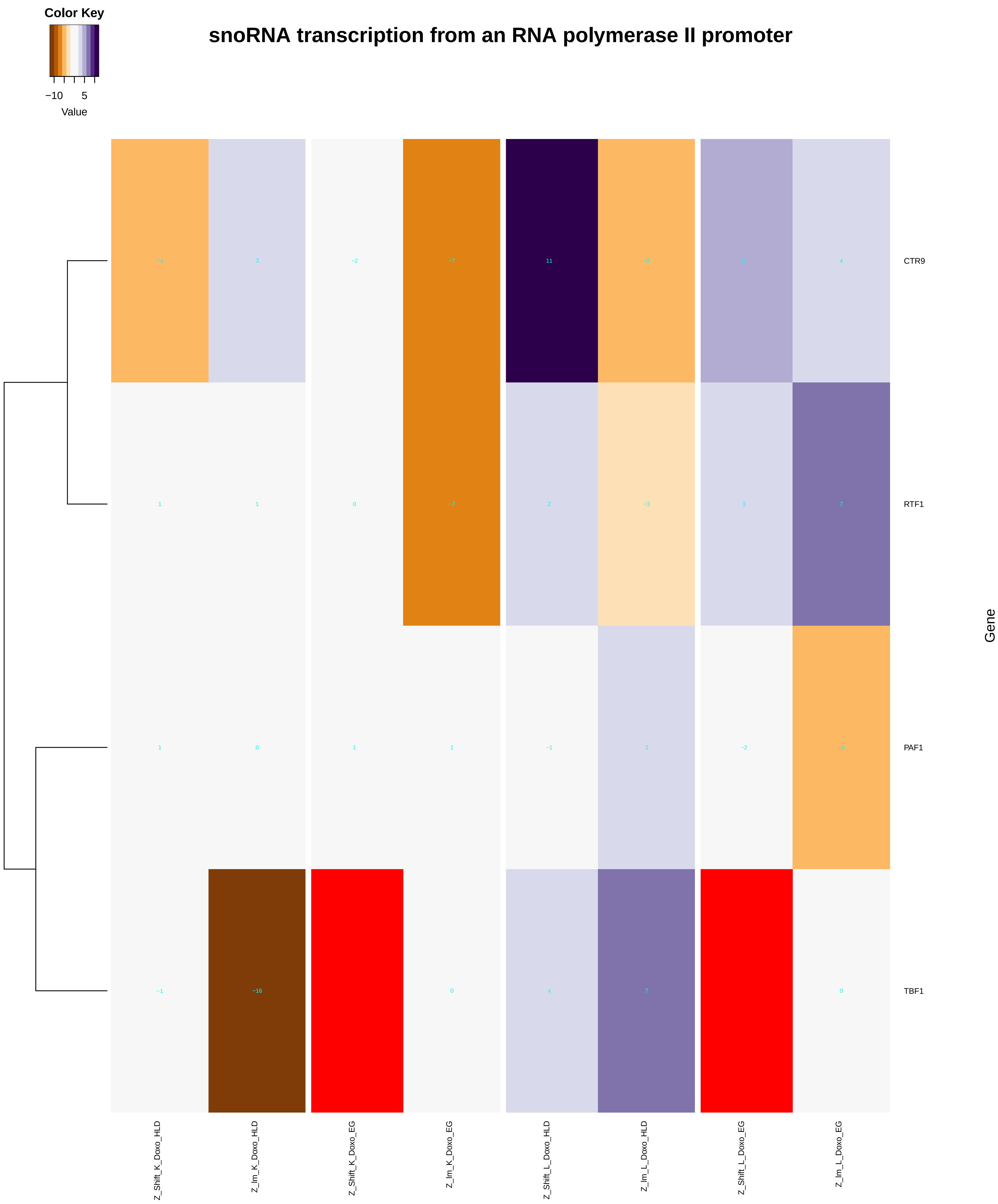

Color Key
−10
5
Value
snoRNA transcription from an RNA polymerase II promoter
CTR9
RTF1
PAF1
TBF1
Gene
Z_Shift_K_Doxo_HLD
Z_Im_K_Doxo_HLD
Z_Shift_K_Doxo_EG
Z_Im_K_Doxo_EG
Z_Shift_L_Doxo_HLD
Z_Im_L_Doxo_HLD
Z_Shift_L_Doxo_EG
Z_Im_L_Doxo_EG

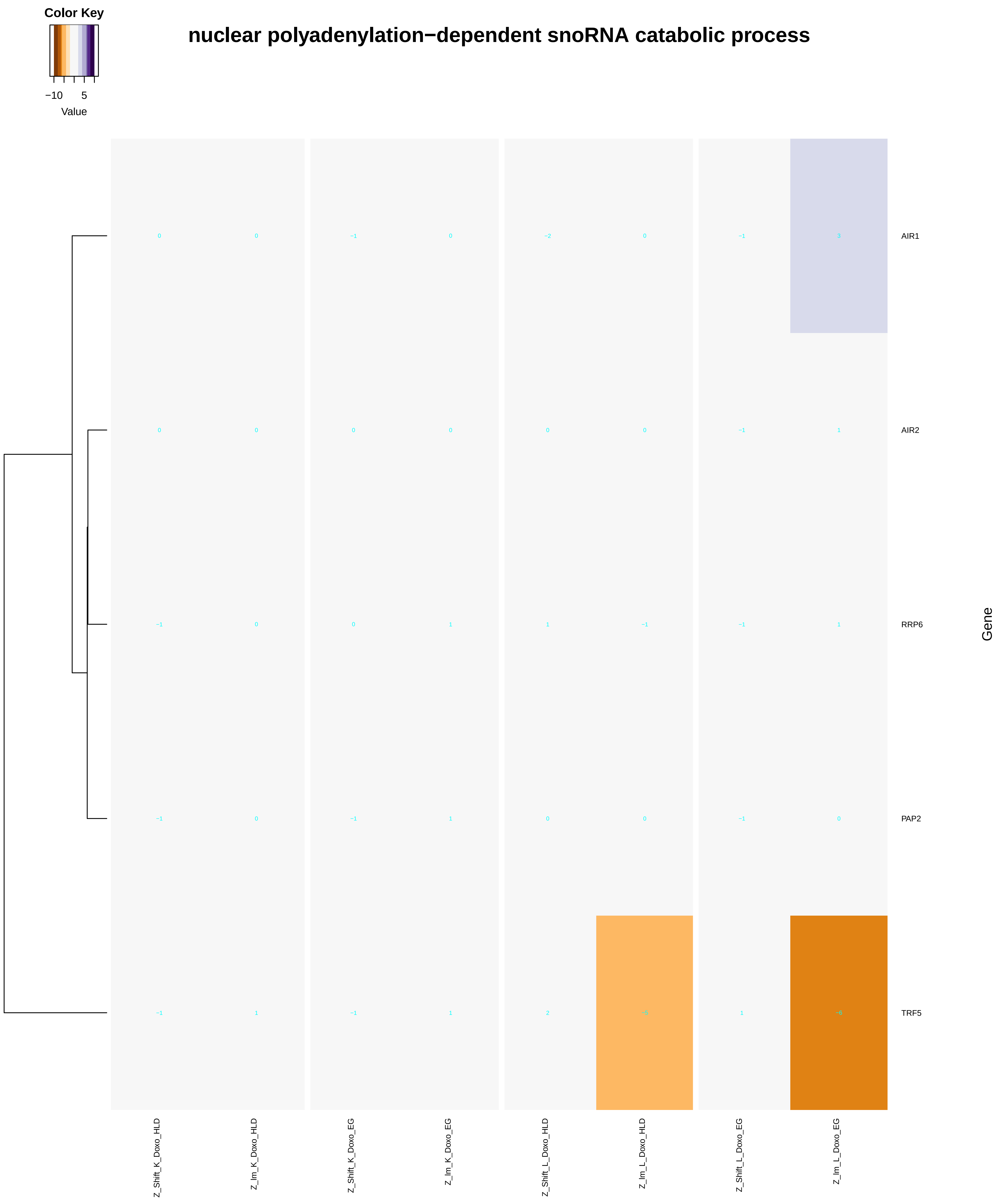

Color Key
−10
5
Value
nuclear polyadenylation−dependent snoRNA catabolic process
AIR1
AIR2
RRP6
PAP2
TRF5
Gene
Z_Shift_K_Doxo_HLD
Z_Im_K_Doxo_HLD
Z_Shift_K_Doxo_EG
Z_Im_K_Doxo_EG
Z_Shift_L_Doxo_HLD
Z_Im_L_Doxo_HLD
Z_Shift_L_Doxo_EG
Z_Im_L_Doxo_EG

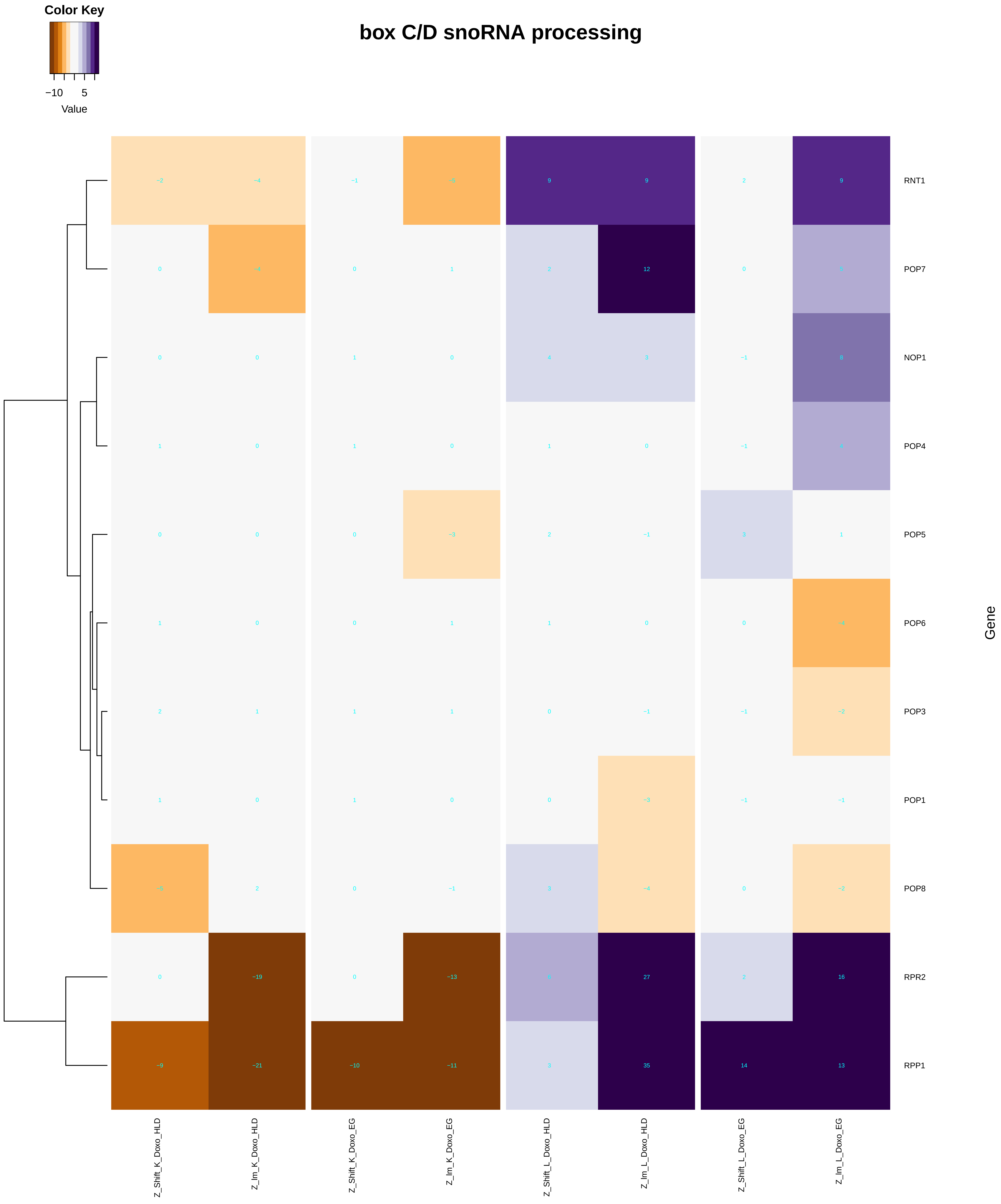

box C/D snoRNA processing
Color Key
−10
5
Value
RNT1
POP7
NOP1
POP4
POP5
POP6
POP3
POP1
POP8
RPR2
RPP1
Gene
Z_Shift_K_Doxo_HLD
Z_lm_K_Doxo_HLD
Z_Shift_K_Doxo_EG
Z_lm_K_Doxo_EG
Z_Shift_L_Doxo_HLD
Z_lm_L_Doxo_HLD
Z_Shift_L_Doxo_EG
Z_lm_L_Doxo_EG

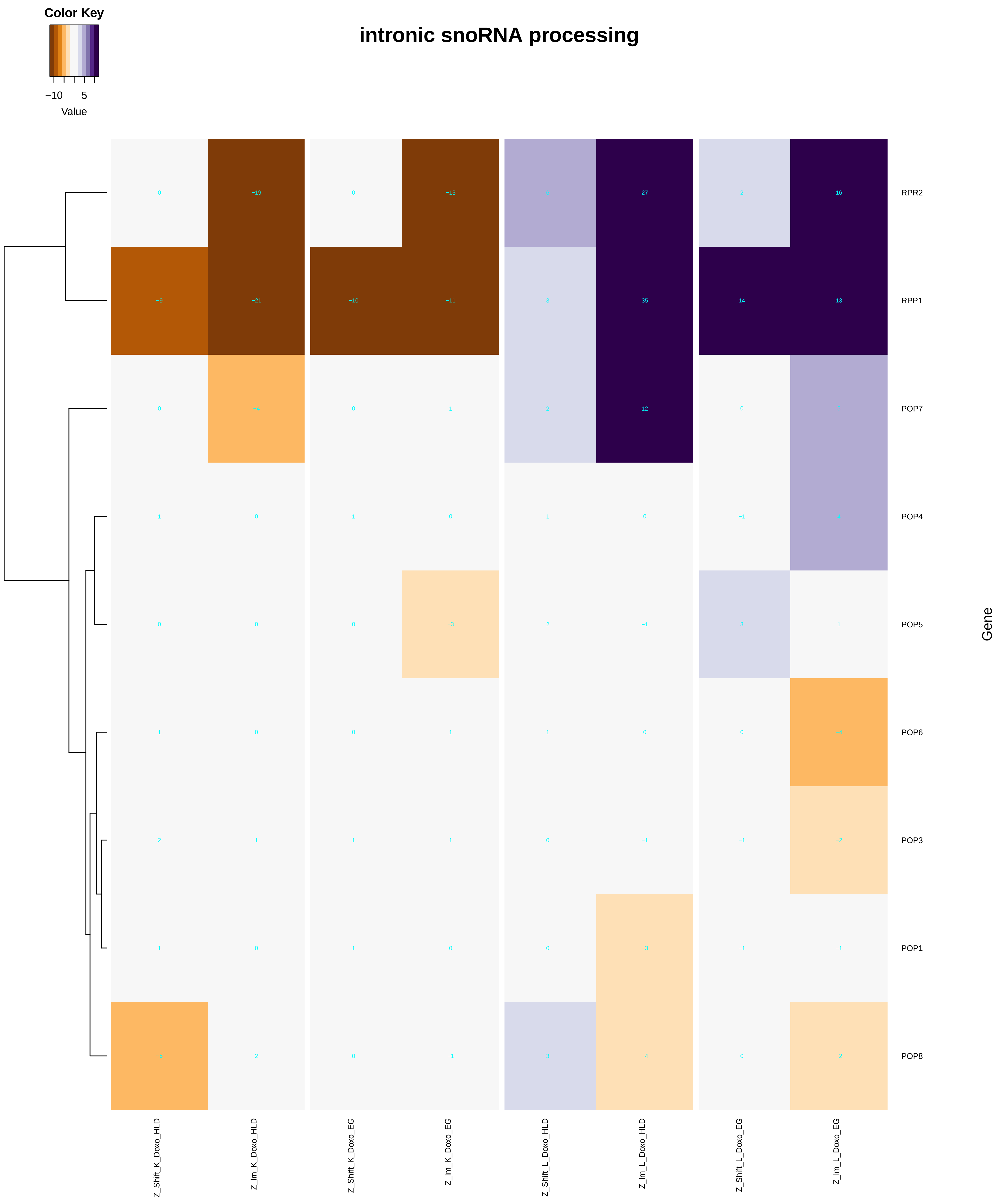

intronic snoRNA processing
Color Key
−10
5
Value
RPR2
RPP1
POP7
POP4
POP5
POP6
POP3
POP1
POP8
Gene
Z_Shift_K_Doxo_HLD
Z_lm_K_Doxo_HLD
Z_Shift_K_Doxo_EG
Z_lm_K_Doxo_EG
Z_Shift_L_Doxo_HLD
Z_lm_L_Doxo_HLD
Z_Shift_L_Doxo_EG
Z_lm_L_Doxo_EG

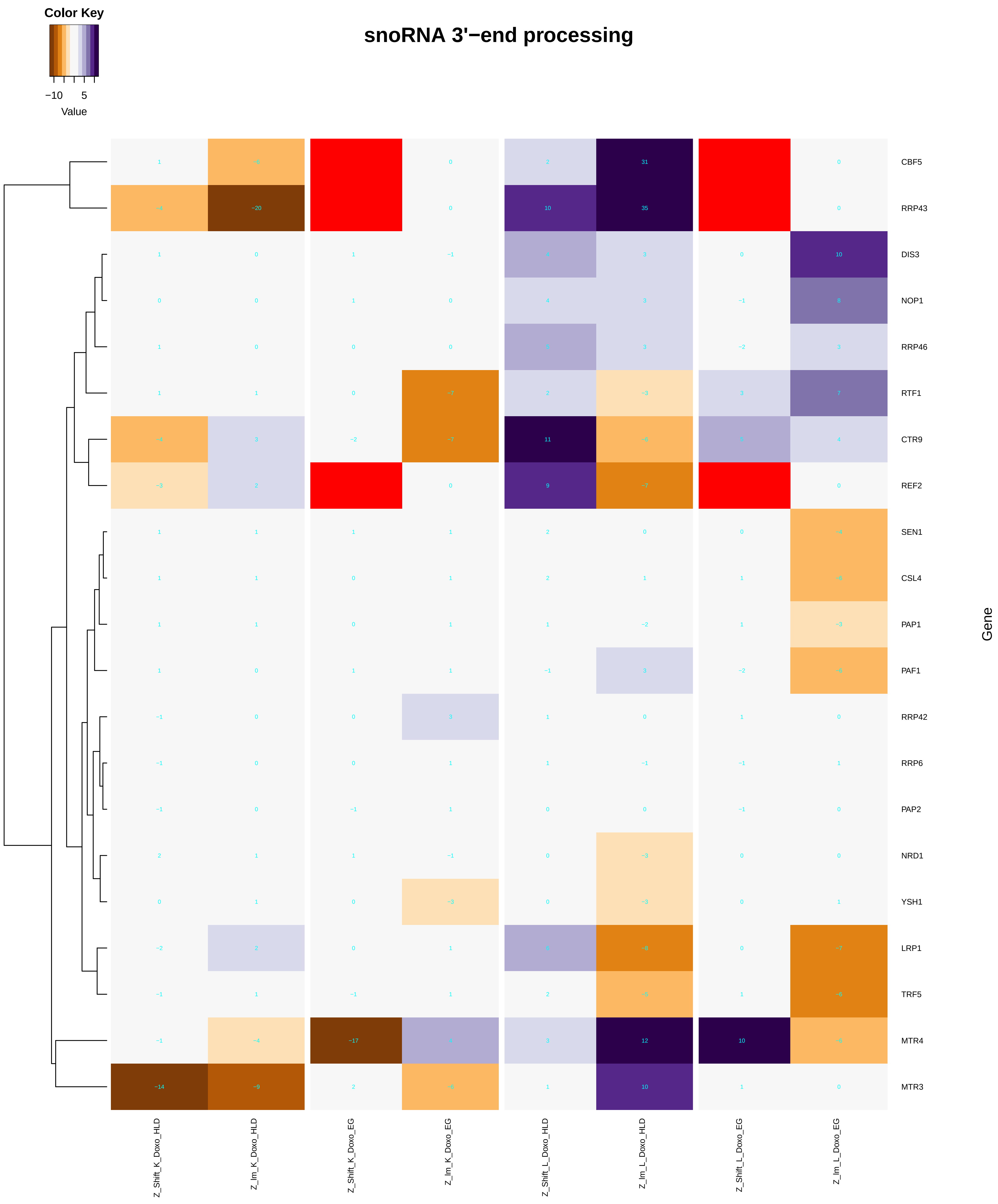
snoRNA 3'−end processing
Color Key
−10
5
Value
CBF5
RRP43
DIS3
NOP1
RRP46
RTF1
CTR9
REF2
SEN1
CSL4
PAP1
PAF1
RRP42
RRP6
PAP2
NRD1
YSH1
LRP1
TRF5
MTR4
MTR3
Gene
Z_Shift_K_Doxo_HLD
Z_Im_K_Doxo_HLD
Z_Shift_K_Doxo_EG
Z_Im_K_Doxo_EG
Z_Shift_L_Doxo_HLD
Z_Im_L_Doxo_HLD
Z_Shift_L_Doxo_EG
Z_Im_L_Doxo_EG

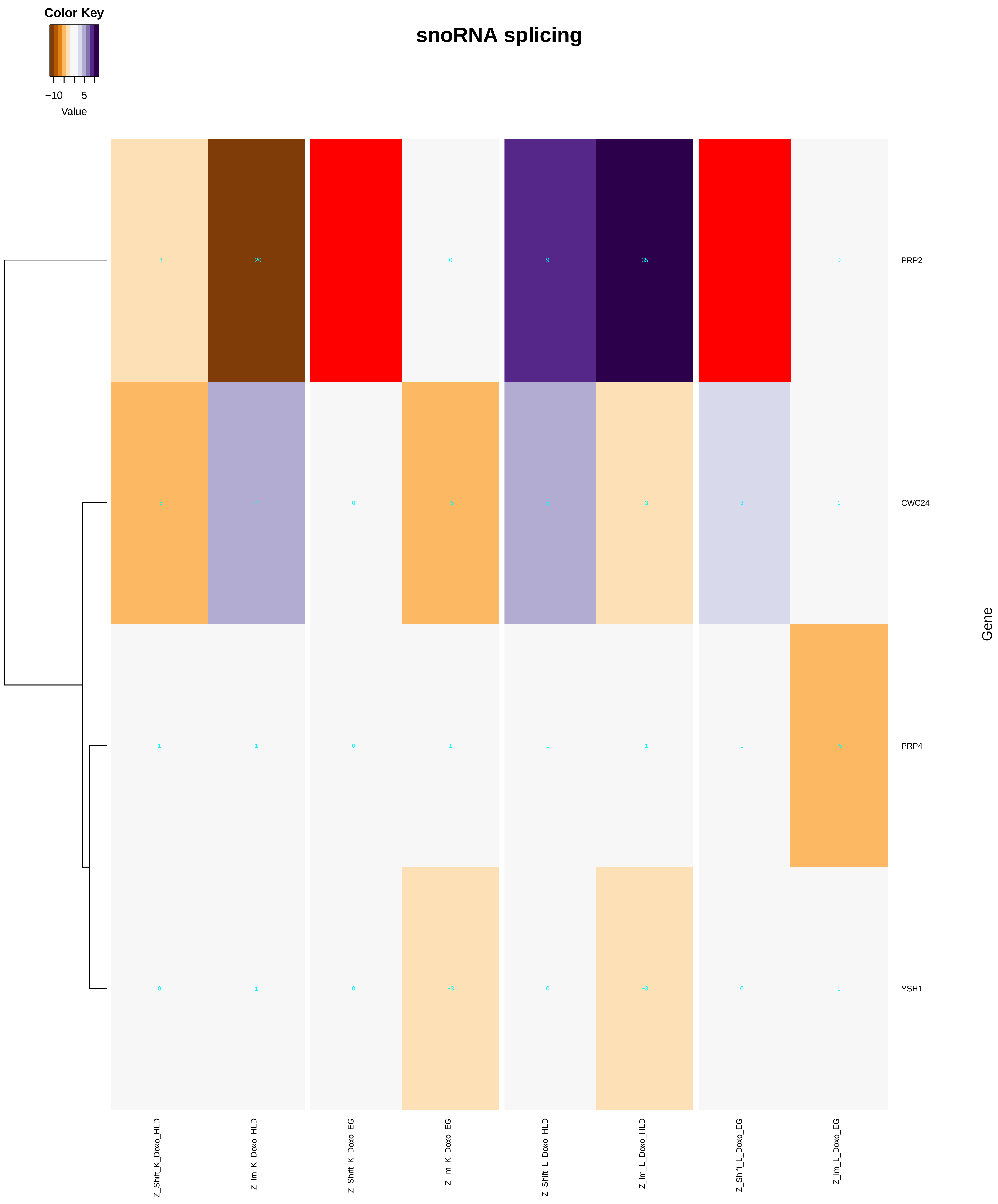
Color Key
−10
5
Value
snoRNA splicing
PRP2
CWC24
PRP4
YSH1
Gene
Z_Shift_K_Doxo_HLD
Z_Im_K_Doxo_HLD
Z_Shift_K_Doxo_EG
Z_Im_K_Doxo_EG
Z_Shift_L_Doxo_HLD
Z_Im_L_Doxo_HLD
Z_Shift_L_Doxo_EG
Z_Im_L_Doxo_EG

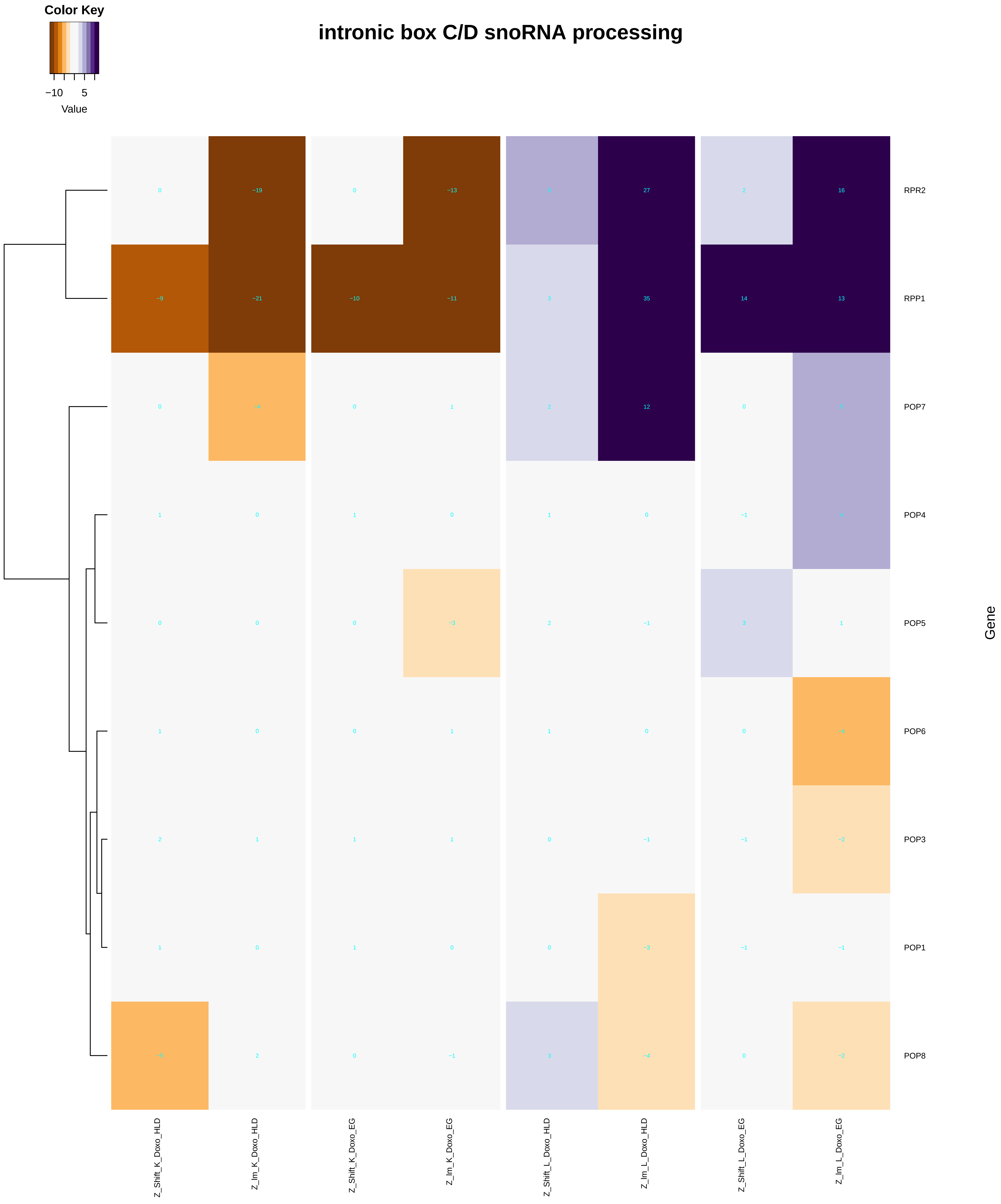
Color Key
−10
5
Value
intronic box C/D snoRNA processing
RPR2
RPP1
POP7
POP4
POP5
POP6
POP3
POP1
POP8
Gene
Z_Shift_K_Doxo_HLD
Z_lm_K_Doxo_HLD
Z_Shift_K_Doxo_EG
Z_lm_K_Doxo_EG
Z_Shift_L_Doxo_HLD
Z_lm_L_Doxo_HLD
Z_Shift_L_Doxo_EG
Z_lm_L_Doxo_EG

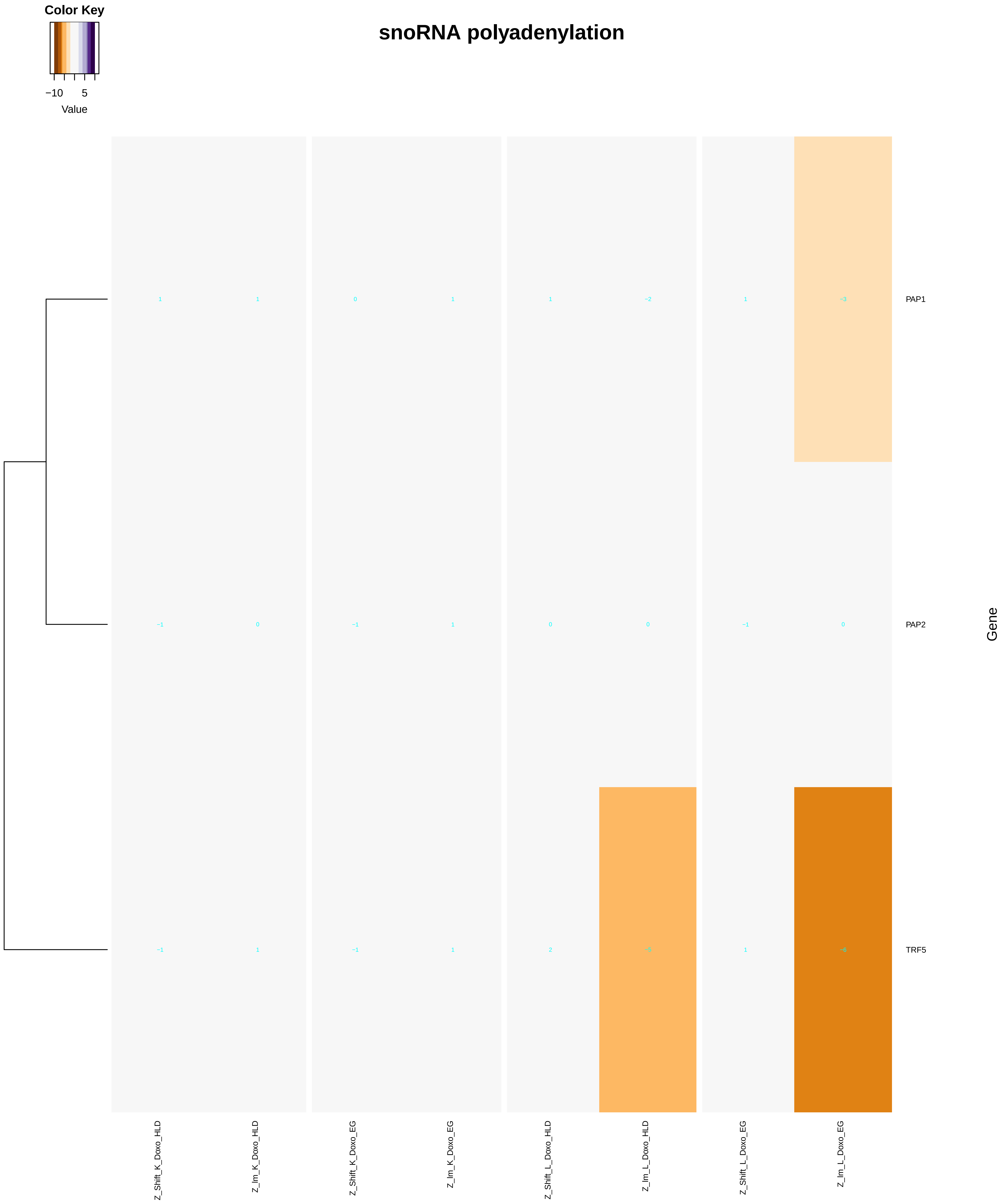

Color Key
−10
5
Value
snoRNA polyadenylation
1
1
0
1
1
−2
1
−3
PAP1
−1
0
−1
1
0
0
−1
0
PAP2
−1
1
−1
1
2
−5
1
−6
TRF5
Gene
Z_Shift_K_Doxo_HLD
Z_Im_K_Doxo_HLD
Z_Shift_K_Doxo_EG
Z_Im_K_Doxo_EG
Z_Shift_L_Doxo_HLD
Z_Im_L_Doxo_HLD
Z_Shift_L_Doxo_EG
Z_Im_L_Doxo_EG

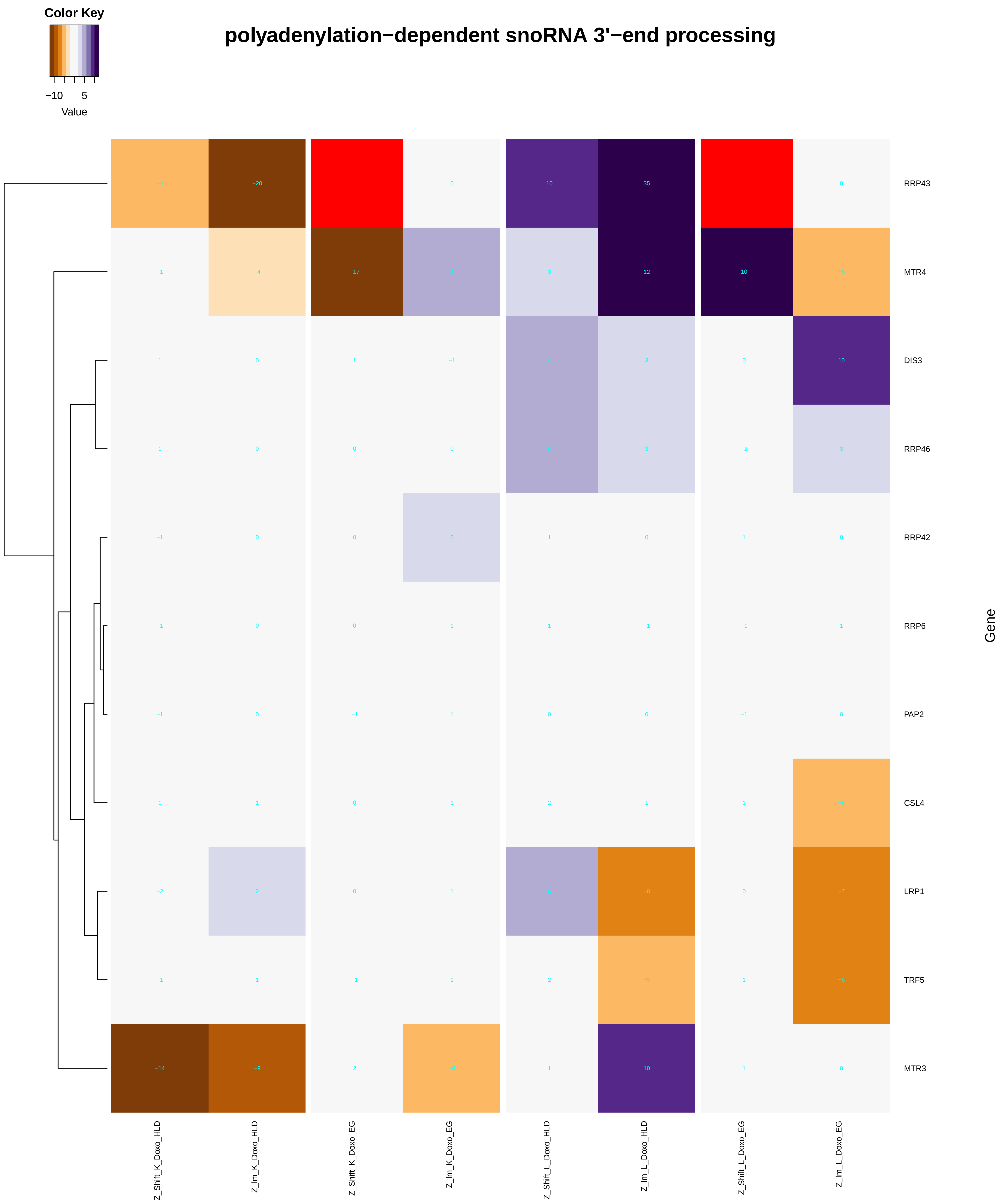
Color Key
−10
5
Value
polyadenylation−dependent snoRNA 3'−end processing
RRP43
MTR4
DIS3
RRP46
RRP42
RRP6
PAP2
CSL4
LRP1
TRF5
MTR3
Gene
Z_Shift_K_Doxo_HLD
Z_lm_K_Doxo_HLD
Z_Shift_K_Doxo_EG
Z_lm_K_Doxo_EG
Z_Shift_L_Doxo_HLD
Z_lm_L_Doxo_HLD
Z_Shift_L_Doxo_EG
Z_lm_L_Doxo_EG
